# Safety and Health Protection of Health Care Workers during the COVID-19 Pandemic

**DOI:** 10.30476/IJCBNM.2020.86066.1319

**Published:** 2020-10

**Authors:** Shahpar Bagheri, Shahram Ghobadimoghadam

**Affiliations:** 1 Community Based Psychiatric Care Research Center, Department of Nursing, School of Nursing and Midwifery, Shiraz University of Medical Sciences, Shiraz, Iran; 2 Department of Nursing, Moharrary Psychiatric Hospital, Shiraz University of Medical Sciences, Shiraz, Iran

Dear Editor

The coronavirus disease 2019 (COVID-19) emerged in Wuhan, China, in late 2019. The fact that COVID-19 has caused illness resulting in death has sustained human-to-human spread, is concerning. The disease has a high mortality rate of between 3 and 15%. ^1^ At the time of writing, the **coronavirus** COVID-19 is transmitted widely among populations and has affected 216 **countries** with more than 5,934,936 cases globally and almost 367,166 deaths. ^2^ Rapid global spread of this new virus is a major public health concern in the world. ^2^ Therefore, healthcare workers, governments and managers need to co-operate to fight against COVID-19 pandemic and it has become increasingly important to address the safety and health protection of healthcare workers. 

Healthcare workers are under both physical and psychological pressure due to exposure to this huge infectious public health problem. ^3^ As of 8 April 2020, 22,073 cases of COVID-19 among health workers from 52 countries had been reported to the World Health Organization(WHO)(2). The role of health workers is vital; they are at the frontline of any outbreak response. They save lives while encountering the hazards that put them at risk of infection with COVID-19 outbreak pathogen. The hazards include physical and psychological violence, long working hours, emotional reactions, fatigue, occupational burnout, stigma, shortage of personal protective equipment, concern about infecting themselves and their family members, insomnia, depression, and anxiety. ^3 , 4^

Therefore, it is imperative to ensure protection for medical staff from hazards not only to safeguard the patients, but also to ensure their health and safety. Hence, the safety and health problems of medical staff in COVID-19 outbreak have become an important public health concern. ^4 , 5^

Evidence suggests implementation of a series of safety and psychological protective measures by managers. Employers and managers in health facilities must take overall responsibility to ensure that all necessary preventive and protective measures are taken to recognize problems and hazards and assess the risks of health workers’ health and safety. They are required to provide information, instruction and training on occupational safety and health, provide adequate personal protective equipment supplies (such as N95 masks, goggles, and protective gowns) in sufficient quantity, reasonable work shift arrangements, sufficient logistical support and comfortable accommodations to healthcare staff during this COVID-19 pandemic. Employers and managers in health facilities should also provide access to mental health professionals and counselling resources to relieve the symptoms in healthcare workers who experience depression, anxiety and psychological distress and seek to recover them from occupational hazards. ^3 - 5^

In conclusion, the managers and employers are suggested to be more sensitive about the health problems with which the medical staff might encounter. Therefore, implementing a series of preventive and protective measures and psychological supportive interventions to ensure the safety and health in healthcare workers could be helpful to overcome this disaster.
